# The onset of ulcerative colitis upon *Helicobacter pylori* eradication in a 72-year-old woman: report of a rare case with a 3-year follow-up

**DOI:** 10.1186/s12876-021-01876-5

**Published:** 2021-07-31

**Authors:** J. Homolak, M. Nikolić, D. Potoč, M. Živković, D. Bakula, I. Budimir, I. Pavić, D. Hrabar, N. Ljubičić, D. Vražić

**Affiliations:** 1 Department of Pharmacology, University of Zagreb School of Medicine, Zagreb, Croatia; 2 Gastroenterology and Hepatology Unit, University Hospital Centre “Sestre Milosrdnice”, Vinogradska 29, 10000 Zagreb, Croatia; 3 University of Zagreb School of Dental Medicine, Zagreb, Croatia; 4 University of Zagreb School of Medicine, Zagreb, Croatia; 5 Department of Pathology, University Hospital Centre “Sestre Milosrdnice”, Zagreb, Croatia; 6 Department of Periodontology, University of Zagreb School of Dental Medicine, Zagreb, Croatia

**Keywords:** Case report, *Helicobacter pylori*, Ulcerative colitis, Inflammatory bowel disease, Golimumab

## Abstract

**Background:**

Epidemiological studies suggest an inverse association between *H. pylori* infection/exposure and inflammatory bowel disease prevalence/incidence, however, there are no reports of individual patients who developed a "non-transient” ulcerative colitis (UC) following *H. pylori* eradication.

**Case presentation:**

We report a case of a 72-year-old female with an elderly-onset UC developed upon *H. pylori* eradication and a 3-year follow-up of the progression to steroid-dependent colitis complicated with enteropathic arthritis and final containment of the disease with golimumab. In our patient, *H. pylori* eradication was associated with the development of pancolitis that evolved into clinically, endoscopically, and pathohistologically confirmed UC.

**Conclusions:**

The case of our patient provides a unique clinical context for a growing body of literature suggesting molecular mechanisms involved in the interaction of genes, environment, and microbiota to be of critical importance in the etiopathogenesis of UC, and thus, provides a valuable set of complementary translational information for preclinical and epidemiological research on the topic.

## Background

*Helicobacter pylori* (*H. pylori*) is an important human pathogen with 50–90% prevalence depending on the stage of economic development [1]. *H. pylori* causes active gastritis associated with peptic ulcers, atrophic gastritis, gastric adenocarcinoma, and MALT lymphoma [2]. Furthermore, it is associated with unexplained iron deficiency anemia, idiopathic thrombocytopenic purpura, vitamin B12 deficiency and there is some early evidence for atherosclerosis, stroke, Alzheimer's and Parkinson's disease [2]. Increasing understanding of the serious pathophysiological consequences of *H. pylori* infection led to a general recommendation published in the Kyoto global consensus report that even individuals without clinically manifest disease should undergo eradication [3]. Interestingly, a growing body of research also reported a negative association of *H. pylori* and some diseases such as asthma [4] and inflammatory bowel disease (IBD) [5], however, the causality of these associations, their clinical relevance, and mechanisms of action are yet to be elucidated.

IBD is an umbrella term representing a group of chronic inflammatory diseases of the gastrointestinal tract. Ulcerative colitis (UC) and Crohn's disease (CD) are the most important clinical entities of IBD characterized by episodes of mucosal inflammation accompanied by abdominal pain, diarrhea, bloody stools, and weight loss [6]. In UC, the disease usually affects the rectum and the part of the colon, but in severe cases, the entire colon can be inflamed. Pathohistological findings usually reveal inflammation limited to the mucosa and submucosa with cryptitis and crypt abscesses [6], and molecular studies indicate increased TH1 and/or TH17 responses accompanied by enhanced production of tumor necrosis factor α, interleukin 1-β, interferon γ, interleukin-17, interleukin-6, and IL-23 [7]. Diagnosis of IBD is usually done by colonoscopy combined with pathohistological examination of colonic biopsies, however exciting new diagnostic approaches such as virtual computed tomography (CT) colonoscopy (CT colonography) [8] and diffusion-weighted imaging techniques [9] have also been proposed recently. Etiopathogenesis of UC is still unknown and recent evidence suggests pathophysiological processes arise as a consequence of a complex interaction of genetic predisposition, dysbiosis, environmental and immunological factors [6].

An inverse association between *H. pylori* infection/exposure and prevalence/incidence of IBD has been reported by many epidemiological studies [5, 10–12]. A comprehensive meta-analysis on the prevalence of *H. pylori* infection in IBD (including UC subgroup) and non-IBD controls suggested a negative association after accounting for ethnicity, age, detection methods, and previous use of drugs (e.g. aminosalicylates and corticosteroids) [5]. Furthermore, an inverse association between *H. pylori* prevalence and disease severity was reported [13]. Several mechanisms have been proposed for the potential protective effect. It has been shown that Hp(2–20), an *H. pylori* ribosomal peptide reduces inflammation and upregulates mucosal damage repair in a rat model of colitis [14, 15]. Microbiota, an important player in IBD [16] is modulated by *H. pylori* [17]. *H. pylori* has been shown to reduce the gastric secretion of the proinflammatory satiety hormone leptin (increased in UC), and increase tolerogenic dendritic cells and suppressive regulatory T cells through IL-18 [5].

Although accumulating evidence suggests *H. pylori* might provide some beneficial anti-inflammatory effects, the results should be interpreted with caution. Observational retrospective studies are susceptible to bias, and appropriate adjustments are not always possible. The incidence of IBD is associated with economic development and "westernization” e.g. increased use of antibiotics, hygiene, cigarette smoking and consumption of processed foods, and decreased family size [18]. The aforementioned factors are also inversely associated with the *H. pylori* infection rates, so the observed association might "merely be a proxy for the *hygiene hypothesis*” [18].

Interestingly, in contrast to extensive epidemiological studies, case reports of patients developing IBD upon *H. pylori* eradication that would provide a “proof-of-concept” are scarce. Jovanovic reported an onset of CD in a 28-year-old upon *H. pylori* eradication [19]. Tursi described a 34-year-old man and a 39-year-old woman who developed CD upon *H. pylori* eradication [20]. Nagami reported a patient requiring a subtotal colectomy following a relapse of UC associated with *H. pylori* eradication [21, 22], and Chiba reported a case of a 63-year-old developing a transient UC upon eradication therapy [22]. To the best of our knowledge no published reports describe the course of *H. pylori* eradication therapy-induced UC that required initiation of biological therapy to achieve satisfactory disease control.

**Fig. 1:**
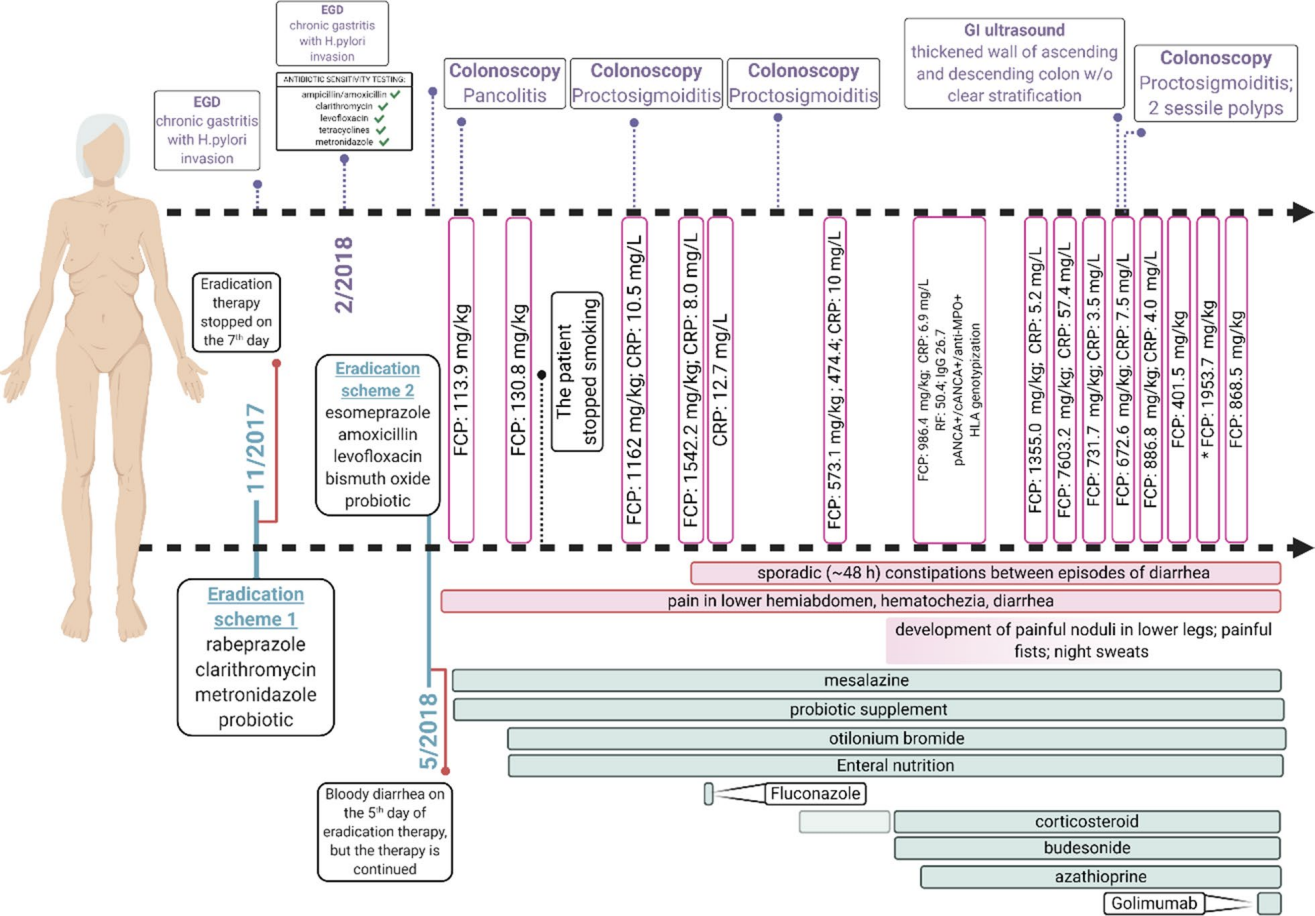
A schematic representation of the case report. FCP—fecal calprotectin; CRP—c-reactive protein

We report a case of a 72-year-old woman who developed UC upon *H. pylori* eradication with a 3-year follow-up, and final containment of the disease following initiation of golimumab (Fig. 1).

## Case presentation

**Fig. 2:**
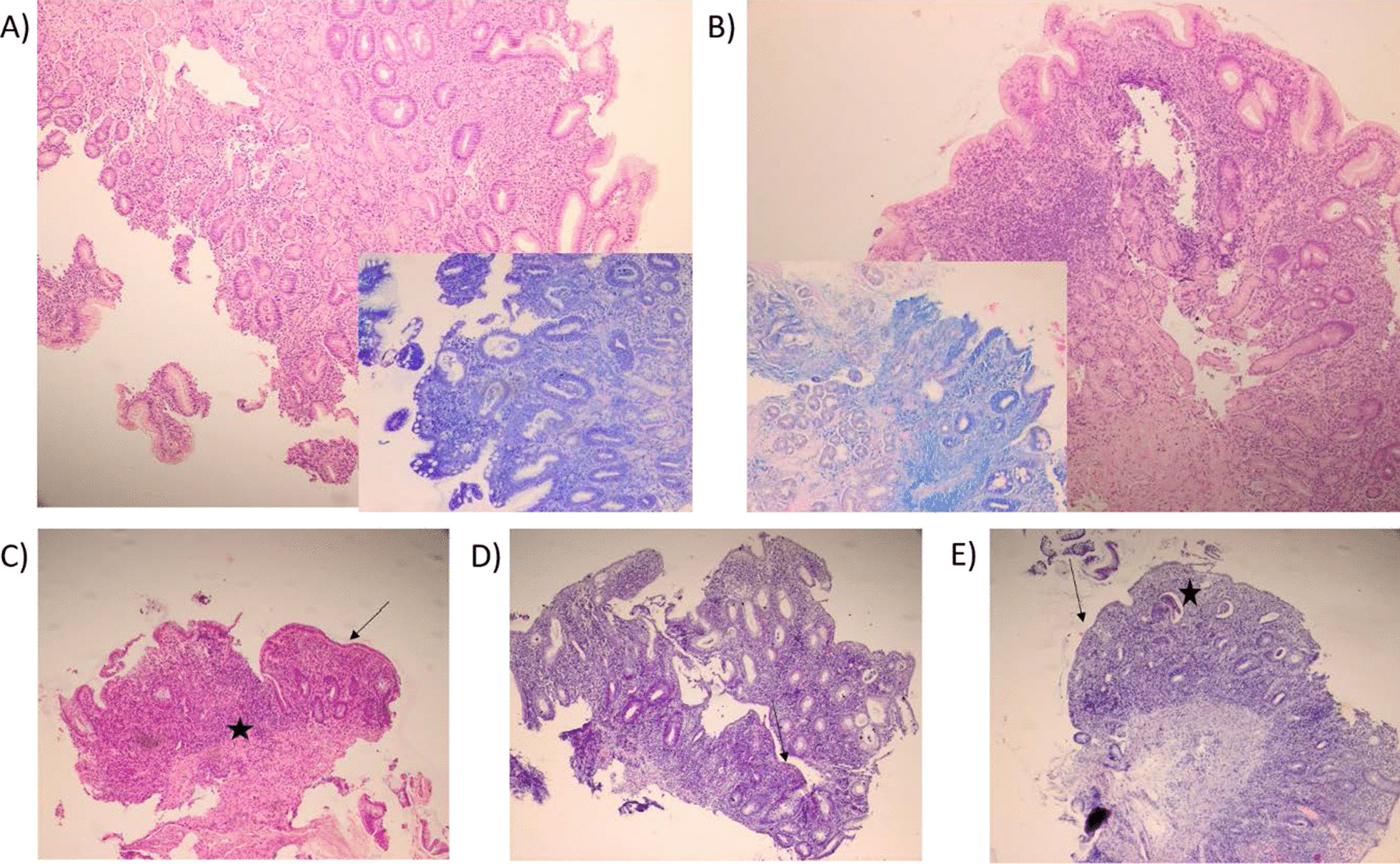
Pathohistological examination of gastric and colon biopsy specimens. **A** Gastric mucosal biopsy taken in 2016 [H&E × 100]; moderate chronic active gastritis with incomplete intestinal metaplasia. Inset: [Giemsa × 100]; moderate *H. pylori* invasion. **B** Gastric mucosal biopsy taken in 2017 [H&E × 100]; moderate chronic active gastritis with incomplete intestinal metaplasia. Inset [Giemsa × 100]; moderate *H. pylori* invasion. **C** Colon mucosal biopsy performed in 2018 after completion of the 2nd attempt of *H. pylori* eradication [H&E × 100]; moderate to severe diffuse inflammatory infiltrate; reduction in colonic gland number (black star); Crypt abscesses present, surface mucous production depletion, and reduced columnar epithelium (black arrow). **D** A follow-up colon mucosal biopsy [H&E × 100]; moderate to severe diffuse inflammatory infiltrate; slight reduction in colonic gland number; surface mucous production depletion and reduced columnar epithelium (black arrow). **E** Colon mucosal biopsy performed in 2020 [H&E × 100]; moderate to severe diffuse inflammatory infiltrate; surface ulcerations, a slight reduction in colonic gland number; surface mucous production depletion and reduced columnar epithelium (black arrow), gland destruction (black star)

A 72-year-old Caucasian female (height-161 cm; weight-82 kg; BMI-31.6 kg/m^2^) presented with a year-long history of dyspeptic symptoms in 2017. The patient reported she was diagnosed with chronic gastritis and gastroesophageal reflux disease in 2016 (Fig. 2A). Past medical history included untreated osteoporosis diagnosed in 2005, thyroidectomy (toxic multinodular goiter in 2013 and a history of Hashimoto's thyroiditis), hypertension, and Reinke's edema-induced hoarseness. Family history and physical examination were unremarkable. The patient admitted to a 50-pack-year of cigarette smoking. She was regularly taking levothyroxine, rabeprazole, amlodipine, and reported penicillin allergy.

Esophagogastroduodenoscopy (EGD) and pathohistology were suggestive of moderate chronic active gastritis with incomplete intestinal metaplasia and *H. pylori* invasion in concordance with findings from 2016 (Fig. 2B). Eradication therapy with rabeprazole, clarithromycin, metronidazole, and a probiotic was initiated. The patient was encouraged to stop smoking.

Seven days in eradication, the patient reported to the emergency department due to redness and tongue swelling. The symptoms were resolved with intravenous methylprednisolone and intramuscular chloropyramine. Eradication was discontinued.

Two months later the patient underwent EGD with biopsy samples subjected to *H. pylori* antibiotic sensitivity testing. Penicillin allergy was ruled out with intracutaneous testing. A combination of esomeprazole, amoxicillin, levofloxacin, bismuth oxide, and a probiotic was proposed for eradication.

Five days in eradication, the patient developed bloody diarrhea but remained highly motivated to continue with the regimen despite the adverse drug reactions (ADRs). Upon successful completion of eradication, colonoscopy was done due to persistent bloody diarrhea. Edematous mucosa of the entire colon and internal hemorrhoids (grade I/II) were revealed and pathohistology was in concordance with pancolitis (Fig. 2C). *C.difficile* (*Clostridioides difficile* toxin A/B Enzyme-linked Fluorescent Assay VIDAS kit (bioMérieux, Marcy l'Etoile, France); detection limit: toxin A [7.73 ng/ml], toxin B [4.55 ng/ml]) and O&P exam were negative. Fecal calprotectin (FCP) was 113.9 mg/kg. The patient was prescribed a light dietary regimen, mesalazine (oral + enema). On the 10th day, a follow-up colonoscopy revealed persistent pancolitis and the patient reported diffuse pain in the lower hemiabdomen, hematochezia, and diarrhea. Successful eradication of *H. pylori* was confirmed with a stool antigen test. The patient was encouraged to remain adherent to the dietary regimen and therapy.

After 2 months, the patient reported a reduction in symptom severity in the period of therapeutic adherence, however, diarrhea persisted despite therapy. In a period of noncompliance, she noticed traces of blood in the stool that disappeared upon a continuation of the recommended therapy. Bacteriological, *C.difficile,* and stool O&P remained unremarkable, and FCP was 130.8 mg/kg. Otilonium bromide and meal replacement were added to the therapy. The patient stopped smoking.

After 4 months, symptom progression was reported: 3 watery stools/day with traces of blood that partially resolved with increased mesalazine. Esomeprazole was added to therapy. Bacteriological, *C.difficile*, O&P, and *H. pylori* remained unremarkable. FCP increased to 1162 mg/kg and c-reactive protein (CRP) was 10.5 mg/L. Colonoscopy revealed proctosigmoiditis, 1.5 cm flat cecal polyp, and several individual diverticula (Fig. 2D). Immunosuppressive therapy was recommended, but the patient declined due to fear of ADRs. Fluconazole was initiated as mycological stool testing revealed abundant *Candida spp*.. CRP was 8.0 mg/L and carcinoembryonic antigen was 6.2 ng/ml. The patient reported watery stools began to alternate with ~ 48 h-long periods of constipation. Pain in the lower hemiabdomen was persistent.

Two months later, a follow-up colonoscopy confirmed proctosigmoiditis (Fig. 2E). Corticosteroid treatment was recommended and a reduction in symptom severity was reported over the next two months although the patient admitted to variable compliance.

Three months later, the patient reported the development of painful shin nodules, pain in her wrists, and increased night sweats. The reported symptoms were considered a sign of enteropathic arthritis and methylprednisolone was initiated. Budesonide was added to therapy and celecoxib, denosumab, and vitamin D were initiated due to osteoporosis. Laboratory findings revealed FCP 986 mg/kg; CRP 6,9 mg/l; rheumatoid factor 50.4 IU/ml; IgG 26,7 g/l; pANCA (1:5120); cANCA (+); anti-MPO 103.9 IU/ml; negative *M.tuberculosis* quantiferon test (0.01 IU/ml). HLA-typization indicated no relevant TPMT mutations and azathioprine was included in therapy.

Clinical improvement was observed after two months. Painful indurations regressed and the patient reported no pain in her wrists. There was 1–2 stools/day with sporadic traces of blood and mild diarrhea. The disease was now partially controlled with a combination of corticosteroid, mesalazine, and azathioprine, however, initiation of golimumab was recommended to avoid complications of steroid-dependent UC.

Golimumab therapy was initiated with a starting dose of 200 mg (sc.) followed by additional 100 mg sc. 2 weeks later, and 100 mg sc. maintenance dose planned every month. So far the patient reported symptom improvement. Corticosteroid and azathioprine were successfully discontinued without a rebound. The disease is currently controlled with a combination of golimumab and mesalazine.

## Discussion and conclusions

We present a case of an elderly woman developing UC upon *H. pylori* eradication with a 3-year follow-up of the progression of the disease that eventually evolved into steroid-dependent UC complicated with enteropathic arthritis that was finally contained with golimumab. Initially, the patient's main concern was mild dyspepsia accompanied with EGD and PHD suggestive of chronic active gastritis with *H. pylori* invasion. Eradication was an obvious next step based on current guidelines [2]. Unfortunately, our patient suffered from ADRs and the therapy had to be stopped after 7 days. Eradication therapy ADRs are relatively common and expected [23], so a second eradication attempt was planned 2 months later after penicillin allergy and *H. pylori* antibiotic sensitivity testing to plan the optimal treatment strategy [24]. Unfortunately, bloody diarrhea developed on the 5th day, and colonoscopy revealed pancolitis that was initially considered either antibiotic-associated or hemorrhagic. Nevertheless, as bloody diarrhea persisted even after the therapy termination, the patient reported a relief following initiation of mesalazine, colonoscopy and PHD indicated UC, and other differential diagnoses were ruled out (e.g. *C.difficile* fecal toxin A and B, O&P)—the patient was diagnosed with ulcerative proctosigmoiditis. Interestingly, a similar pattern of events was observed in a 63-year-old who developed bloody diarrhea upon *H. pylori* eradication therapy that was initially understood as antibiotic-associated colitis, but the condition didn't resolve on its own following treatment discontinuation [22]. Although both patients developed UC upon treatment with the *H. pylori* eradication therapy, several differences should be emphasized. In [22], eradication therapy-induced UC was transient and resolved within 3 months following 24 days of 750 mg/kg/day metronidazole [22]. Furthermore, although the UC-like signs and symptoms appeared in parallel with the *H. pylori* eradication regimen, a subsequent urea breath test indicated the infection was still present. As further information regarding *H. pylori* presence was not reported, the patient might have been *H. pylori* positive throughout transient UC. In contrast, *H. pylori's* absence was confirmed in our patient. Furthermore, UC severity and clinical course were less favorable for our patient. Fortunately, the disease is currently under control with golimumab, a reassuring finding considering the benefit of golimumab maintenance therapy was previously observed in patients who responded to golimumab induction [25, 26].

The presented case speaks in favor of the inverse association between *H. pylori* infection/exposure and prevalence/incidence of IBD proposed by numerous studies [5, 10–13, 27–33]. The majority of studies reporting an inverse relationship between *H. pylori* and IBD are ill-equipped to provide a mechanistic explanation for the observed effects. Nevertheless, recent evidence from preclinical experiments suggest chronic *H. pylori* infection may protect against IBD by inducing systemic immune tolerance and suppressing inflammatory responses [7]. For example, *H. pylori* infection reduces systemic inflammation and splenic CD4^+^ T cells and ameliorates colitis-associated histopathological changes in the dextran sulfate sodium-induced mouse model of colitis. Furthermore, in mice infected with *Salmonella typhimurium*, a co-infection with *H. pylori* increased mesenteric lymph node interleukin-10 and prevented the TH17 response [7]. In a recent comprehensive review of *H. pylori*-induced immunomodulation activation of dendritic cell tolerogenic phenotype and immunosuppressive regulatory T cells were proposed as the most important mechanisms mediating potentially beneficial effects in IBD [7].

Interestingly, some studies reported no association between *H. pylori* and UC [34], and some even proposed it may act as a causative agent triggering the development of the disease. For example, Mansour et al. analyzed colonic biopsies from 30 patients newly diagnosed with UC and 30 controls and found *H. pylori* in 56.6% of cases and 20% of controls using polyclonal anti-*H. pylori* antibodies [35].

One possible explanation of the apparently paradoxical findings of the protective/harmful effects of *H. pylori* in IBD is that the differences in seroprevalence between IBD and the controls are a consequence of pharmacotherapy. For example, the protective role of 5-aminosalicylic acid on *H. pylori* infection has been proposed [36]. Similarly, some authors suggested other drugs such as metronidazole, corticosteroids, sulfasalazine or quinolones could also be responsible for the observed differences [7]. Nevertheless, several studies found no effect of IBD drugs on *H. pylori* seroprevalence [7].

## Strengths and limitations

Strengths: (1) long-term follow-up; (2) defined early course of the disease (pancolitis evolving to proctosigmoiditis) as colonoscopy of the only other patient [22] reported 40 days after eradication suggested proctosigmoiditis; (3) differential diagnoses were ruled out (eg. bacteriological and O&P stool examinations); (4) *H. pylori* eradication was confirmed; (5) biochemical indicators of disease severity were monitored and reported.

Limitations: We cannot rule-out that (1) our patient would develop UC regardless of the *H. pylori* eradication (~ 20% of patients do after the age of 60 [37]); (2) UC onset was treatment and not *H. pylori* related. Although the risk of autoimmune diseases (including IBD) is increased in patients with peptic ulcer disease (PUD) who receive anti-*H. pylori* therapy, the same is true for PUD patients who didn't receive therapy and for patients who received antibiotics for urinary infection [38]. Antibiotics have been suggested both as risk factors and potential therapy for IBD. A recent systematic review on antibiotic exposure and risk of IBD suggested there might be an association [39], and a large population-based retrospective cohort indicated increased risk in subjects exposed to antianaerobic antibiotics [40]. On contrary, many randomized controlled trials reported antibiotics to be effective for induction of UC remission [41] and several meta-analyses suggest overall higher remission rates with antibiotics in comparison with placebo [42, 43]. The fact that the patient stopped smoking as well as that she had underlying thyroid disease should also be acknowledged in the context of UC as both are potential factors that could have affected the course of the disease. Smoking was found to be protective in UC, decreasing the need for colectomy in some patients possibly due to the effect on cellular and humoral immune responses and mucus production [44]. Furthermore, it has been reported that autoimmune disorders are more frequent in patients with IBD indicating possibly overlapping pathophysiological processes and/or shared risk factors [45]. We cannot rule out that both factors affected the course of the disease. Nevertheless, smoking cessation could not have caused UC as the patient stopped smoking well after the disease developed. On the other hand, genetic susceptibility to autoimmune diseases could have contributed to the predisposition for subsequent development of UC.

## The primary "take-away”

Taking into account a consensus that all infected individuals should be treated with *H. pylori* eradication therapy, it is to be expected that rare cases of eradication-induced UC might become more prevalent. In our patient, an unfortunate combination of environmental and genetic risk factors (predisposition to autoimmune disorders, e.g. Hashimoto's thyroiditis), complicated with *H. pylori* eradication-induced dysbiosis triggered pathophysiological events leading to the development of a serious case of UC. In the future, a better understanding of the molecular mechanisms responsible for the disease development, factors influencing the protective and/or harmful effects of *H. pylori* (e.g. recent report indicated protective effects of *H. pylori* in IBD are mediated by the cytotoxin-associated gene A positive strain [46], and there is some evidence of mediation by the protective cross-reactivity to IBD aggravating *C.jejuni* [5]), and personalized approach to the *H. pylori* eradication might help inform a new set of guidelines based on maximizing the beneficial effect of eradication, while minimizing the risk of potential side effects of eradication treatment.

## Data Availability

Data will be provided upon reasonable request and in concordance with maximal protection of the patient privacy.

## Abbreviations

*H. pylori*: *Helicobacter pylori*
IBD: Inflammatory bowel disease
UC: Ulcerative colitis
CD: Crohn's disease
CT: Computed tomograpy
EGD: Esophagogastroduodenoscopy
ADRs: Adverse drug reactions
FCP: Fecal calprotectin
CRP: C-reactive protein
PUD: Peptic ulcer disease
